# Chilling temperature remodels phospholipidome of *Zea mays* seeds during imbibition

**DOI:** 10.1038/s41598-017-08904-z

**Published:** 2017-08-21

**Authors:** Agathe Noblet, Juliette Leymarie, Christophe Bailly

**Affiliations:** 1 Sorbonne Universités, UPMC Univ Paris 06, CNRS, Institut de Biologie Paris-Seine (IBPS), UMR 7622, Biologie du développement, F-75005 Paris, France; 2 Present Address: Université Paris Est Créteil, Faculté des Sciences et Technologie IEES Paris, EcoPhyS, 94100 Créteil, France

## Abstract

Global warming is a major agricultural issue in the Northern hemisphere where higher temperatures are expected to be associated with restricted water availability. In Europe, for maize, earlier and further northward sowings are forecasted in order to avoid water deficit periods in the crop life cycle. However these conditions may compromise seed germination and stand establishment since they will take place at cold temperatures. It is urgent to better understand the molecular bases of response of germinating maize seeds to cold in order to design genotypes adapted to these novel agricultural practices. Here we have performed a global phospholipidomic study to profile changes in membrane reorganisation during seed imbibition at 10 °C of cold-tolerant and -sensitive maize hybrids. Using a Multiple Reaction Monitoring (MRM-MS/MS) method coupled with HPLC we have identified 80 distinct phospholipids. We show that seed sensitivity to cold temperatures during imbibition relies on the accumulation of saturated or poorly unsaturated fatty acids, whatever the phospholipid class. In contrast seeds of cold-tolerant hybrid accumulated polyunsaturated chains which was associated with lower electrolyte leakage during imbibition at 10 °C. The expression of fatty acid desaturase genes provides a molecular model of maize seed sensitivity to imbibitional chilling damage.

## Introduction

In Europe maize is the second major crop and it represents almost a quarter of the total cereal production^1^. South France, Italy, Hungary and Romania are the main areas for maize production thus forming a geographic belt where the environmental conditions are optimum for growth and development of this crop. Studies on climate change performed in the last decade show consistent projections of increases in temperatures and changes in precipitation patterns at the global scale^2^. In temperate areas, warming is predicted to increase by 2 °C in 2030 and water stress will dramatically increase in the southern regions of Europe. Both phenomena are expected to greatly reduce maize crop yields especially because they will affect the phenological stages of flowering and grain maturation^3, 4^. Therefore novel agricultural practices must be designed in order to counteract the negative impacts of climate change, while taking advantage of its positive effects. Creation of stress tolerant hybrids and modification of sowing dates, with the use of early maturing hybrids, are short terms options which are already implemented^5^. In addition, land re-allocation is a long-term adaptation which is more and more discussed^6, 7^. Indeed, in Europe, a northern shift of maize production area would allow to prevent more adverse conditions and would permit maize crops not to suffer from water deficit during the critical steps of growing^8, 9^. Although attractive, this strategy will however lead maize seeds to germinate and maize seedlings to grow in inappropriate thermal conditions encountered at the time of stand establishment in European northern regions. Maize is indeed naturally adapted to environmental conditions of tropical regions^5^ and the base temperature (Tb) commonly used for this plant is 10 °C^10^, indicating that a temperature of 10 °C can be defined as “low” or “cold” for this species, as this is stated in this work. In consequence cooler temperatures of European northern areas are expected to greatly affect seed germination and seedling establishment of maize which could in consequence prevent the successful use of re-allocation strategies.

Seed germination is a complex and tightly regulated process which starts with water absorption by the dry seed and ends when radicle elongates^11^. Its achievement requires a synchronized achievement of many cellular processes including DNA repair, protein synthesis or membrane reorganization^11, 12^. In dry seeds membranes are in a gel state and if water enters the seed before their transition to a liquid crystalline state leakage and damage can occur, this process being emphasized at low temperature^13^. Rapid and successful completion of germination is a key stage for the establishment of vigorous seedlings and is also considered as a major component of final crop yield^14^. Germination is very sensitive to the environmental conditions and in particular strongly depends on temperature^13^. In the case of maize, which hardly develops below 10 °C, very few is known about the molecular mechanisms of chilling tolerance or sensitivity during germination, in contrast to the effect of cold on seedlings or at the whole plant level. In the context of crop relocation and climate change it therefore becomes urgent to better understand the effects of low temperature on maize seeds during germination.

Sensitivity of plants to cold stress has often been associated with an uncontrolled accumulation of reactive oxygen species (ROS)^15^. For example ROS have already been shown to accumulate in maize seedlings in response to cold thus causing oxidative stress^16, 17^. Membrane remodeling is also widely acknowledged as a major consequence of cold stress in plants. Low temperatures modulate the phospholipid composition of plasma membrane and more particularly the level of unsaturation of fatty acids^18–20^ which in turn affects properties of fluidity and permeability of biological membranes^21^. Plasma membrane phospholipids formed by two fatty acids esterified on the positions sn-1 and sn-2 of a glycerol, a phosphate on the position sn-3 to which a polar head is linked. Phosphatidylinositol (PI), phosphatidylserine (PS), phosphatidylethanolamine (PE) and phosphatidylcholine (PC) are classes of glycerophospholipids synthesized in the endoplasmic reticulum (ER) whereas phosphatidylglycerol (PG) class is synthesized in chloroplasts. Fatty acid desaturases control the number of unsaturation of acyl chains and are compartment-dependent in plants^21^. Fatty acid biosynthesis starts in the lumen of chloroplasts where SSI2 (formerly called FAB2, Steroyl-Acyl Carrier Protein Desaturase) provides the first unsaturation to saturated 16-C and 18-C carbon, with a strong affinity for stearic acid (18:0)^22^. Then, for PI, PE and PC, FAD2, an endoplasmic-located enzyme, adds a second unsaturation to oleic acid (18:1) whereas PG desaturation occurs in the chloroplast where the homolog plastidial ω-6 desaturase (FAD6) generates the second unsaturation^21^. The third unsaturation in 18-C chains of PI, PE and PC is provided by FAD3 in the ER and by FAD7/FAD8 in the chloroplast for PG^21^.

Since membrane reorganization is one of the major event occurring during seed imbibition, and since cold temperatures are known to modify membrane phospholipids, we have investigated whether sensitivity of maize seeds to low temperature, *i.e*. 10 °C, at the germination stage might rely on changes in the phospholipidome. To address this question, we have used a mass spectrometry (MS)-based lipidomic method. This method has been recently adapted to plant lipid extracts for studying phospholipases D substrates from Arabidopsis^23^. It successfully permitted to analyse the wide range of molecular species that constitute Arabidopsis phospholipids. The technique of mass spectrometry used in MRM (Multiple Reaction Monitoring) associates mass spectrometry with CID (Collision Induced Dissociation)^24^. MRM is particularly adapted to analyse phospholipid composition because it gives access to the identification and quantification of fatty acid couples of each individual phospholipid species. The powerfulness of this technique has been shown by Djafi *et al*.^25^ who demonstrated that it allowed to monitor the changes of phospholipids composition in response to abiotic stress in Arabidopsis, and by Tellier *et al*.^26^ and Zhou *et al*.^27^ who studied *Arabidopsis thaliana* or *Camelina sativa* lipid seed composition.

In the present study we have taken advantage of the MRM method to decipher the molecular changes occurring at the level of membrane organization in seeds of two maize hybrids of different seed vigour during their imbibition at 10 °C.

## Results

### Effect of low temperature on germination of seeds of hybrids A and B

**Figure 1:**
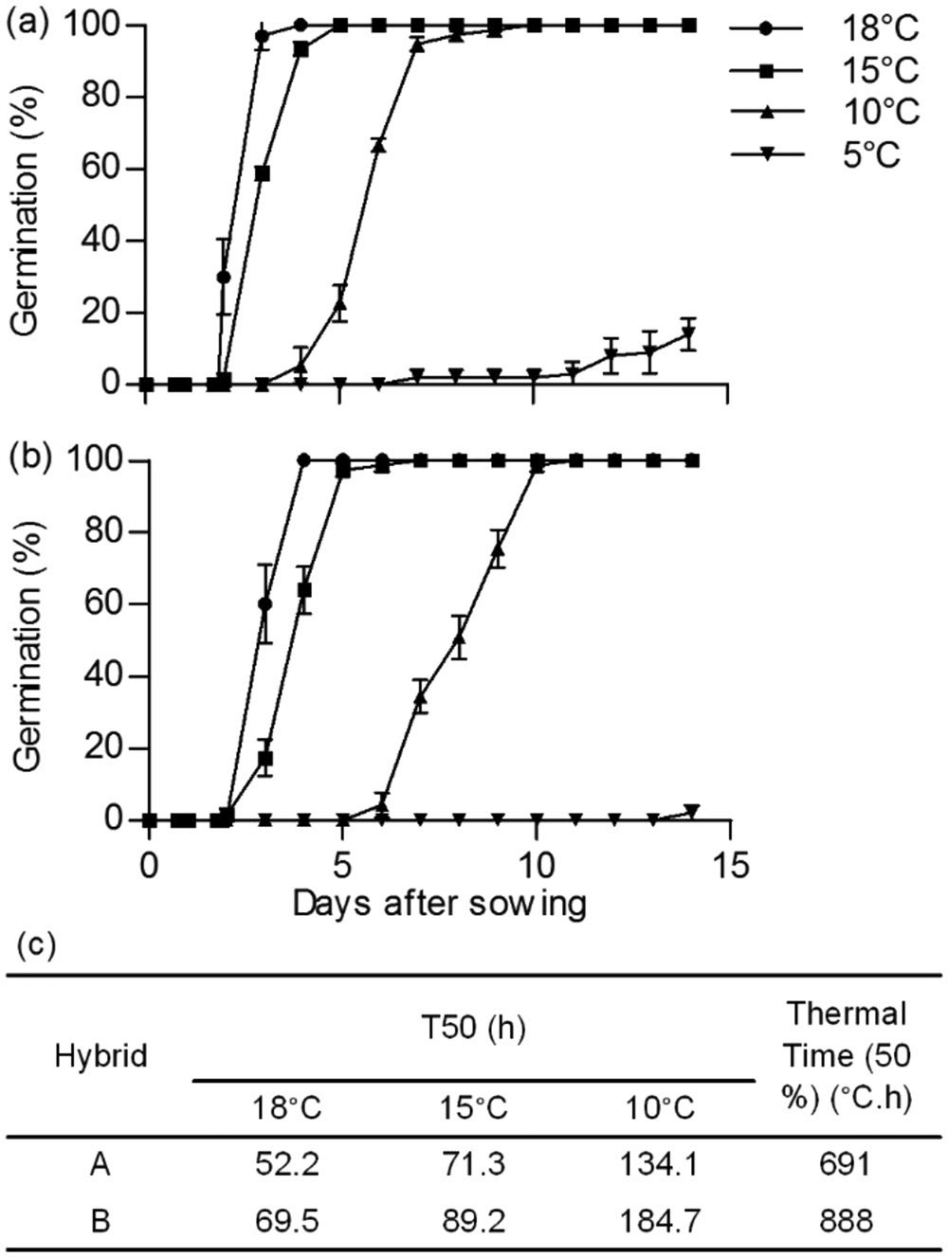
Effect of temperature on seed germination. Germination of hybrids A (**a**) and B (**a**) at 4 different temperatures and cardinal temperatures for germination (**c**). Each point is the mean of 3 replicates of 25 seeds each ± SD.

Maize seeds of hybrids A and B fully germinated at 18 °C within 3 and 4 d, respectively (Fig. 1). Germination was delayed when temperature decreased but it still reached 100% at 10 °C whereas it was strongly inhibited at 5 °C (Fig. 1a and b). The inhibitory effect of cold on germination rate was higher on seeds of hybrid B. For example, they required 10 d to fully germinate at 10 °C, when seeds of hybrid A germinated at 100% within 7 d at this temperature, which was confirmed by the T50 and thermal time values shown in Fig. 1c. At 10 °C the T50 value dramatically increased in hybrid B, when compared to the one estimated at 18 °C, and to the one of hybrid A. In agreement, seeds of hybrid A displayed a smaller thermal time than seeds from hybrid B (Fig. 1c). Altogether these data suggest that seeds of hybrid A are more tolerant to cold temperature during their imbibition than seeds from hybrid B.

### Changes in lipid peroxidation and electrolyte leakage during cold imbibition

**Figure 2:**
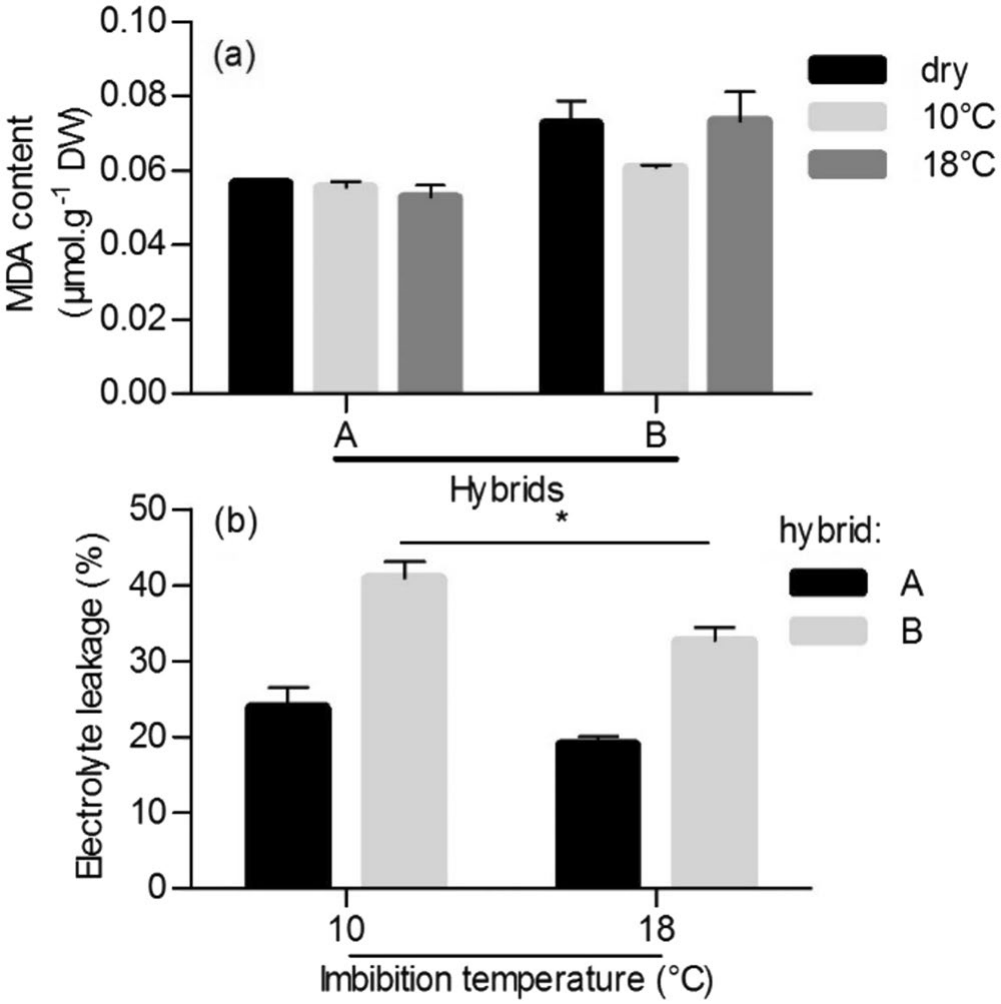
Malondialdehyde (MDA) embryo content (**a**) and electrolyte leakage (**b**) of seeds of hybrids A and B after 24 h imbibition at 10 °C and 18 °C. Means of 3 replicates. Vertical bars correspond to SD. Asterisks above the bar indicate that electrolyte leakage in hybrid B at 10 °C differed significantly from that measured at 18 °C (*P < 0.05).

In order to know whether the differential sensitivity of seeds of hybrids A and B to cold could be related to differential lipid peroxidation or lipid membrane properties, we have measured MDA content, a lipid peroxidation indicator, and electrolyte leakage during imbibition at 10 °C. MDA amount was determined in dry embryos and in embryos imbibed at 10 °C and 18 °C but it was roughly similar in these three conditions for each hybrid, even though seeds of hybrid B always contained more MDA than seeds of hybrid A (Fig. 2a). Electrolyte leakage was measured after 24 h imbibition at 10 and 18 °C (Fig. 2b). Imbibition at 10 °C resulted in a significant increase in electrolyte leakage, when compared to imbibition at 18 °C, but in hybrid B only. It is worth to note that electrolyte leakage was always higher in hybrid B than in hybrid A at 10 and 18 °C (Fig. 2b).

### Effect of cold imbibition on membrane composition

**Figure 3:**
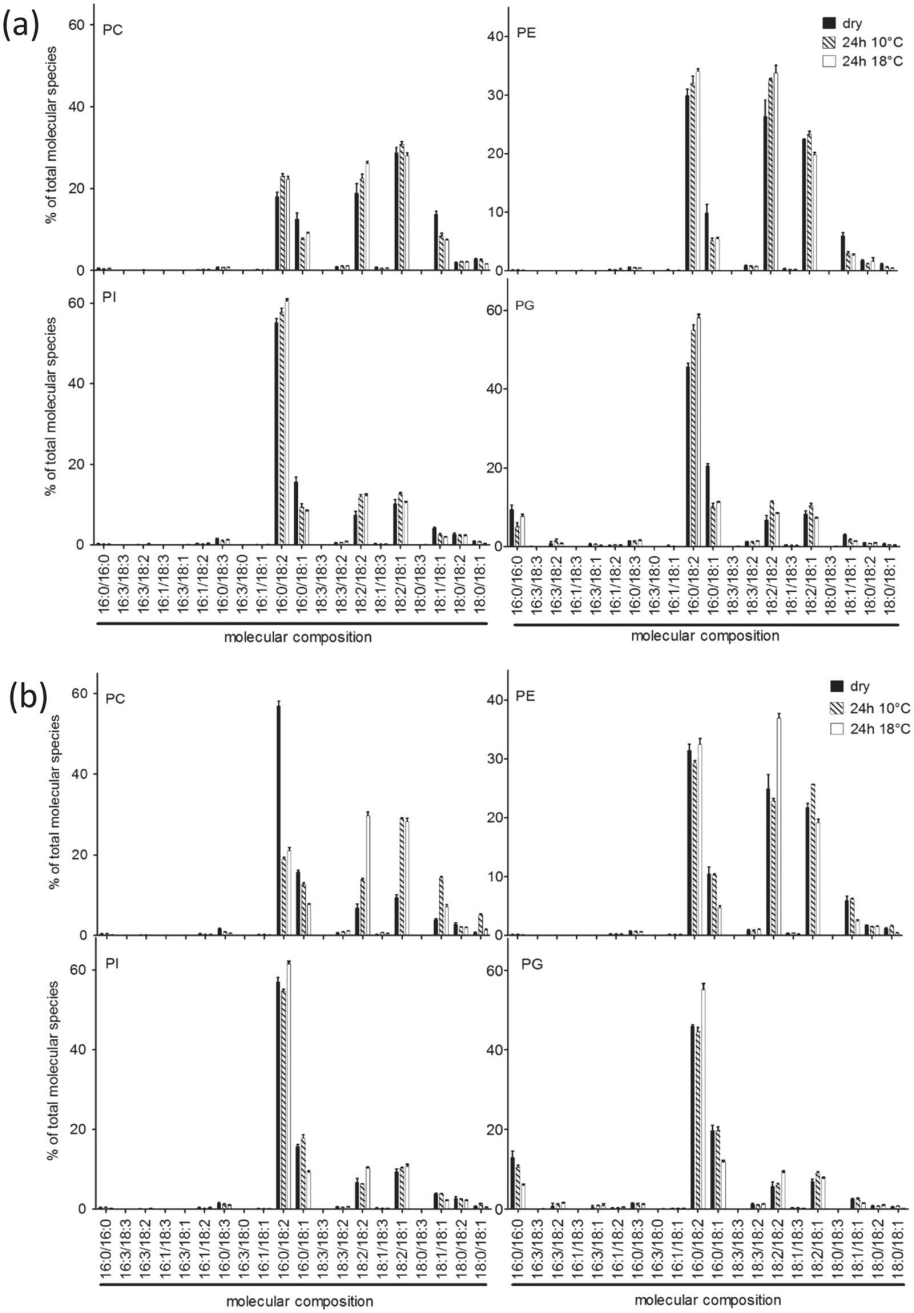
Phospholipid profiling of seeds of hybrids A (**a**) and B (**b**). Molecular species composition of phosphatidylcholine (PC), phosphatidylinositol (PI), phosphatidylethanolamine (PE) and phosphatidylglycerol (PG) extracted from embryos of hybrids A. Composition of fatty acid couples is expressed in percentage of total molecular species in each phospholipid class. Seeds were either dry (black bars) or imbibed at 10 °C (shaded bars) or 18 °C (white bars). Values are means ± SD of 3 triplicates.

With regards to the differences of electrolyte leakage between seeds of hybrids A and B during their imbibition at 10 °C, we performed lipidomic analyses to profile lipid membrane composition of dry embryos and of embryos imbibed for 24 h at 10 °C and 18 °C. The analyses allowed to identify and to relatively quantify 80 different molecular species of 4 phospholipid classes: phosphatidylcholine (PC) (20 species), phosphatidylethanolamine (PE) (20 species), phosphatidylinositol (PI) (20 species) and phosphatidylglycerol (PG) (20 species) (Fig. 3). No signal above noise was detected for digalactosyldiacylglycerol (DGDG) and galactosyldiacylglycerol (MGDG). Figure 3 shows the relative amount of molecular species for the different classes of phospholipids for the 3 conditions, each species being characterized by the couple of acyl chains that composes it. All phospholipids contained C16 and/or C18 acyl chains. PC and PE classes generally displayed a higher proportion of C18 chains than PG and PI. Lipid composition of the two cultivars appeared globally similar for dry seeds (black bars) and for seeds imbibed at 18 °C (white bars). The only significant difference was found in dry seeds, since PC of hybrid B contained more 16:0/18:2 and less 18:2/18:2 and 18:2/18:1 than PC from hybrid A (Fig. 3b). When seeds were imbibed at 10 °C (shaded bars), the esterified chains 16:0/18:2 increased among all classes in hybrid A and decreased in hybrid B. In contrast, the couple 18:1/18:1 decreased in hybrid A and increased in hybrid B, but only in PC. The couple 16:0/18:1 decreased in hybrid A but remained unchanged in hybrid B. Mostly, the changes induced by cold imbibition affected the nature of the couples of acyl chains, regardless their class.

**Figure 4:**
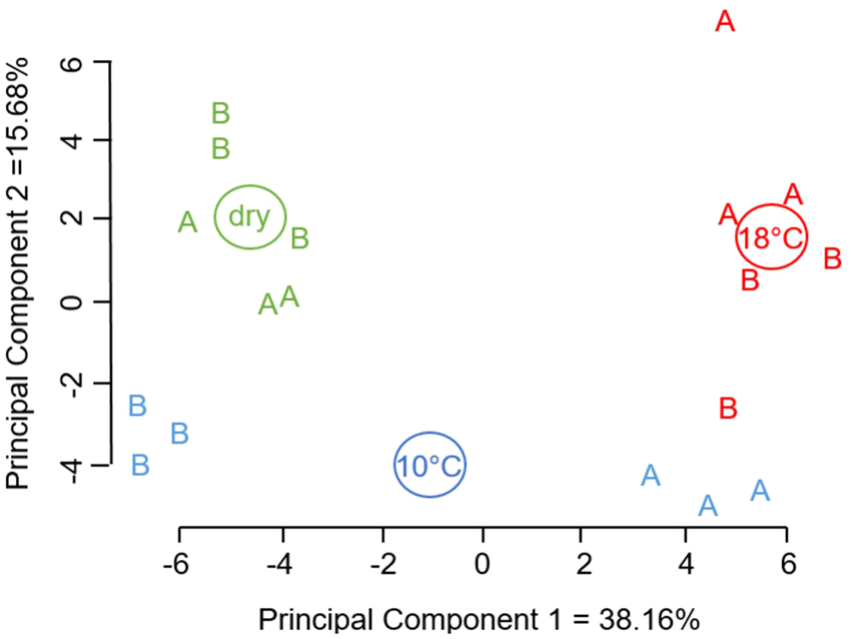
Principal component analysis (PCA) of membrane composition of embryos of hybrids (A and B). Score plot of lipid species content of dry seeds (green) and seeds imbibed for 24 h at 10 °C (blue) or 18 °C (red). Each letter represents a replicate of hybrid (A or B). Principal components 1 and 2 (PC1 and PC2) explain 38.16% and 15.68% of the variance in the data set. Loadings can be found in Supplemental Table S2.

To reveal whether patterns of membrane lipid composition were modified by cold imbibition, a principal component analysis (PCA) has been carried out on the triplicates of the 80 phospholipids species for the two hybrids (Fig. 4). Principal component 1 (PC1) explained 38% of total variance and principal component 2 (PC2) explained 15% of total variance. Given that the two first components explained 53% of the variance overall, score plot has been used to unravel differences and similarities between the 3 conditions (dry, imbibition at 10 and 18 °C) and between hybrids A and B (Fig. 4). Triplicates of dry seeds of the two hybrids plotted together with a negative value for PC1 and a positive for PC2. Triplicates of the 18 °C-imbibed seeds also gathered together with a positive value of PC1 and PC2, but with slight intra- and inter-hybrid heterogeneity. Interestingly the composition patterns of the cold imbibed seeds differed between the two hybrids which were separated by the first principal component (Fig. 4). Regarding the loadings of the two first principal components, PC1 is mainly composed by species containing at least one linoleic acid (C18:2), with apparently no difference among lipid classes (Supplemental Table S1).

**Figure 5:**
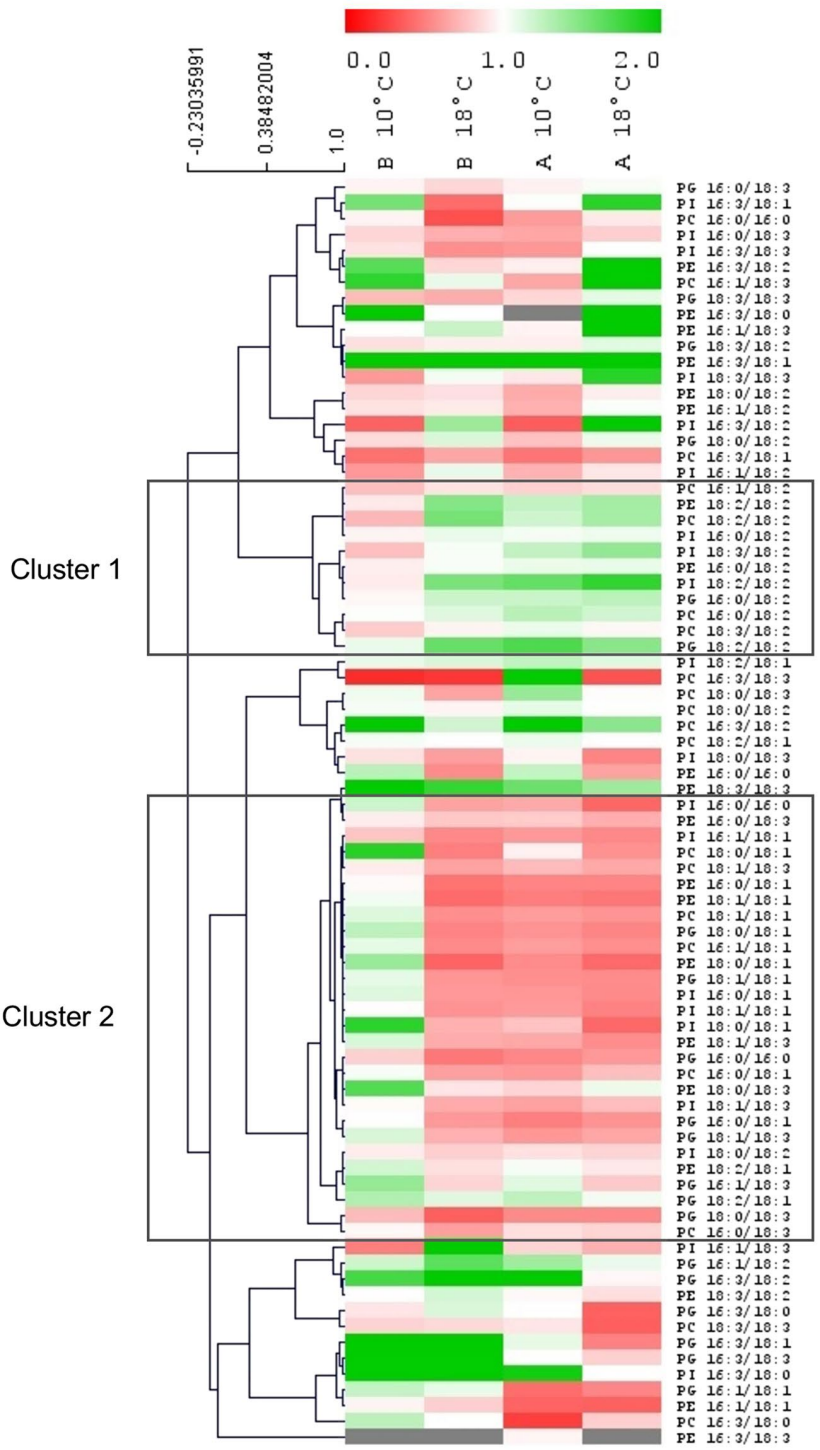
K-mean clustering analysis of lipid species after 24 h imbibition at 10 °C or 18 °C. Each case represents the ratio between phospholipid composition of imbibed to dry embryos. Red is used for ratios < 1, green for ratios > 1 and white for ratio = 1. Clusters 1 and 2 cluster species with reduced or increased ratio in hybrid B when imbibed at 10 °C, respectively.

Changes in the relative content of phospholipids between hybrids are shown on a Heatmap (Fig. 5) where changes are expressed as the ratio of the lipid species relative content in imbibed embryo to that in dry embryo. A K-mean clustering analysis allowed to identify two clusters of species whose ratio at 10 °C in hybrid B was different from both imbibition at 18 °C and hybrid A. Cluster 1 contained molecular species whose content was lower at 10 °C in hybrid B only. Linoleic acid (C18:2) was the most represented fatty acid in this cluster. Cluster 2 contained phospholipids whose amount increased in cold and in hybrid B. It contained species with saturated and mono-saturated fatty acid chains. In addition this analysis confirmed that cold affected the composition of fatty acids without any preference for the phospholipid class.

**Figure 6:**
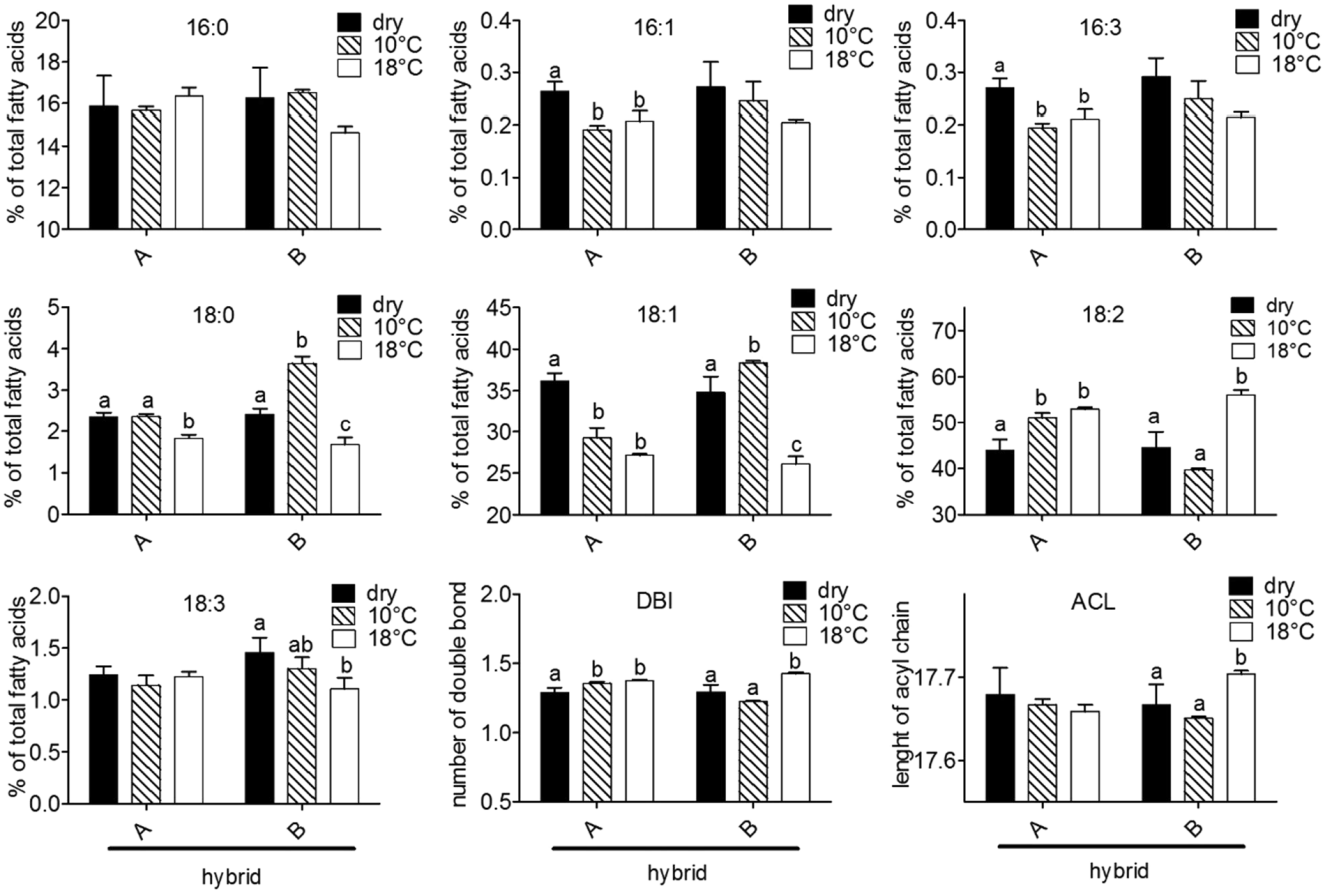
Fatty acid composition in phosphatidylcholine (PC). Proportion (in %) of fatty acid chains in dry and imbibed embryos of hybrids A and B. DBI: double bond index, ACL: acyl carbon length. Different letters denote significantly different mean values at P < 0.05 according to Tukey’s multiple range test. Absence of letters indicates that means are not significantly different.

These results led us to closely investigate the changes in membrane fatty acid composition as a function of the imbibitional temperature. Composition of fatty acid chains have been determined for each lipid class (PC, PI, PE and PG) and results are provided in Fig. 6 and in supplemental data (Supplemental Figures S1, S2 and S3). As an example, Fig. 6 shows the fatty acid composition of PC for both hybrids in the 3 experimental conditions. Regarding C16 acyl chains, seeds of hybrid A contained slightly lower amount of C16:0 at 10 °C whereas proportion of C16:1 and C16:3 was similar in dry seeds of hybrids A and B and dropped down when embryos were imbibed at either 10 or 18 °C (Fig. 6). Interestingly hybrid B accumulated C18:0 and C18:1 chains at 10 °C whereas hybrid A accumulated C18:2 chains in this condition. The double-bond index (DBI) corresponds to the mean number of unsaturation per fatty acid. Imbibition at 10 °C led to a significant increase of DBI in seeds of hybrid A. In hybrid B, the DBI did not differ between dry seeds and seeds imbibed at 10 °C but it was significantly higher when seeds were imbibed at 18 °C. Similar trends were found for the 3 other phospholipid classes (Supplemental Figures S1, S2 and S3) except for seeds of hybrid B which accumulated only C18:1 chains at 10 °C in PI (Supplemental Figure S1) and PG (Supplemental Figure S3). In PE the composition of fatty acids of hybrid B was similar in dry and imbibed seeds whereas hybrid A accumulated 18:2 chains during imbibition at 10 °C (Supplemental Figure S2). For PI species, the acyl carbon length (ACL), which corresponds to the mean number of carbons of fatty acids, was higher in seeds of hybrid A after imbibition at 10 °C when compared to the other conditions (Supplemental Figure S1).

### Expression of desaturase genes

**Figure 7:**
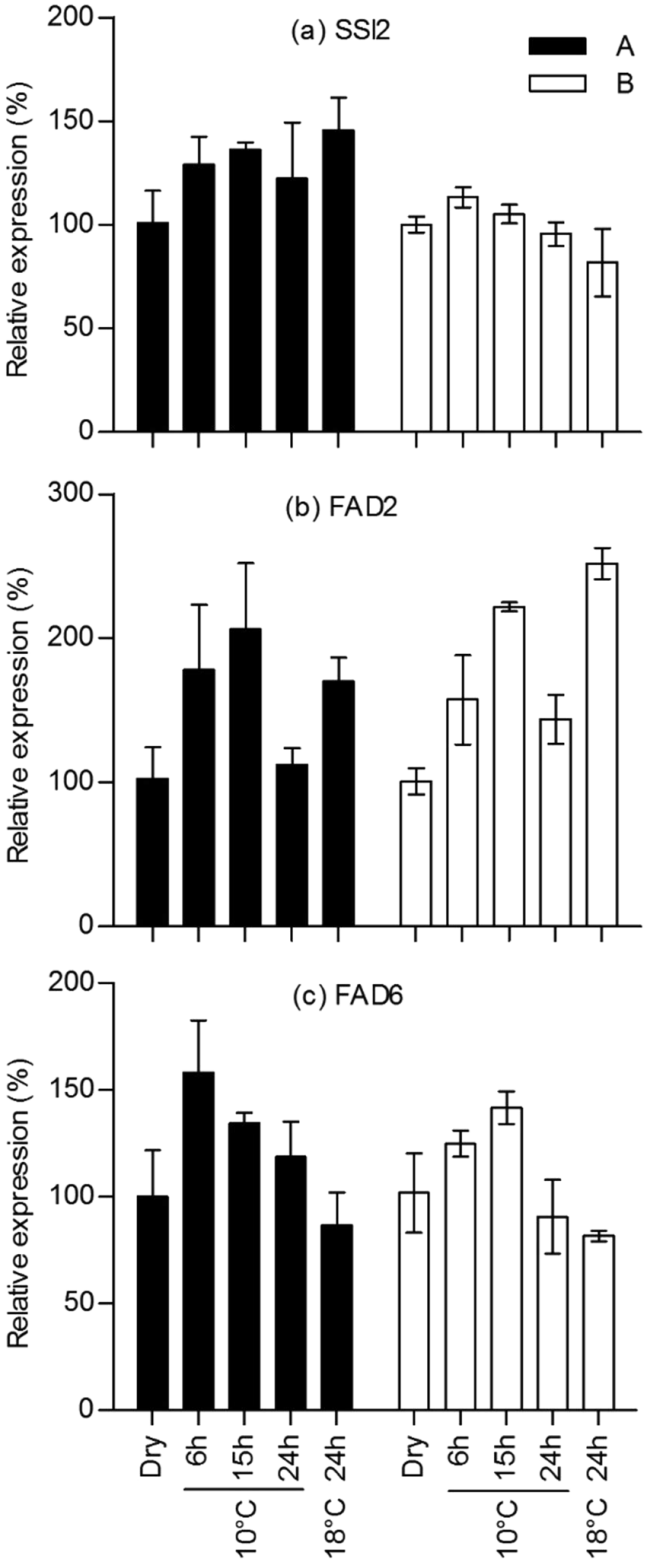
Transcript abundance of desaturase transcripts in maize embryos. Expression of *ssi2* (**a**), *fad2* (**b**) and *fad6* (**c**) in dry embryos of hybrid A (black bars) and hybrid B (white bars) and various durations of imbibition at 10 °C or 18 °C. The relative expression was calculated from qRT-PCR data with 3 references genes, cdk, 2og-fe and unknown, and expressed in arbitrary units with a value of 100 attributed to the dry seeds. Means ± SD of three biological replicates are shown.

Since lipidomic analysis demonstrated that cold imbibition could specifically modify fatty acid (FA) unsaturation levels in seeds of hybrids A and B, we have investigated the changes in the expression of desaturases in order to determine whether this could result from a transcriptional regulation. Expression of *fad3* and *fad7*/*fad8* was not studied here because maize embryos contain very low amounts of C18:3 (Fig. 6 and Supplemental Figures S1, S2 and S3). Using real time quantitative PCR we have quantified the abundance of transcripts of the 3 genes which provide the monosaturated FA (chloroplastic *ssi2*) and bi-saturated FA (endoplasmic f*ad2* and chloroplastic *fad6*) at different time points during seed imbibition at 10 °C and after 24 h at 18 °C (Fig. 7). The mRNA levels of *ssi2* tended to be higher in hybrid A than in hybrid B whatever the temperature of imbibition (Fig. 7a). Expression of *fad2* gene was roughly similar in both hybrids during seed imbibition at 10 °C, but it was always higher at 18 °C in genotype B (Fig. 7b). In seeds of hybrid B, the expression of *fad2* was stimulated by imbibition at both 10 and 18 °C whereas in hybrid A it increased at 18 °C only. At last, seed imbibition at 10 °C was associated with a stimulation of *fad6* expression: the expression peaked after 6 h for hybrid A but only after 15 h for hybrid B (Fig. 7c).

## Discussion

The strategy of maize crop relocation has been designed to address the challenge of global warming. Relocation of maize plants in northern areas is considered for avoiding high temperatures and low precipitations that are predicted to prevail during the summer period in the actual European areas of maize production, thus permitting to maintain high yields. Nevertheless this strategy requires rethinking crop management, especially because seed sowing may thus occur under unfavourable temperatures. This is particularly crucial for maize since low temperatures in northern areas at the time of sowing will negatively impact germination. The present set of data provides a better fundamental knowledge on the effects of cold temperature during the imbibition stage which might be in turn useful for the creation of novel genetics resources displaying adaptive traits through the development of marker assisted selection.

Low temperature (10 °C) during grain imbibition strongly delayed radicle emergence compared to non-penalizing temperature (18 °C) but this temperature was not lethal since grain finally germinated to 100% (Fig. 1). However, the strong reduction of germination rate at this temperature indicates that it can be considered as a stressful temperature^28^. In practice, cultivating crop species in thermal conditions far from the optimum (*e.g*. 10 °C here) can have a dramatic effect on final yield^29^. More particularly slow and heterogeneous seed germination has a direct impact on final yield since rapid seedling emergence is a major component of crop yields^14^. Due to its tropical/subtropical origin, the sensitivity of maize to cold is well known. As it was shown by Seget’a^30^ and various authors (reviewed by Greaves^5^) germination of maize seed is altered at suboptimal temperatures in a genetic dependent manner, which is in agreement with the differential germination rates of hybrids A and B shown here. Nevertheless the genetic variation for germination at cold temperature suggests that comparison of cold sensitive and tolerant hybrids may reveal key traits for this character and that such comparison is very suitable for a better understanding of the mechanisms involved in cold tolerance at the germination stage.

ROS are widely considered as compounds involved in perception and response of plants to a wide range of environmental conditions, including at the germination stage^27^. In maize, the involvement of ROS and lipid peroxidation in response to cold^16, 17, 31, 32^, but also to other abiotic stresses^33, 34^, is well documented at the seedling stage, *i.e*. after radicle emergence had occurred, but to our knowledge not at the germination stage. MDA is considered as a good indicator of oxidative stress in seeds, especially for those with lipid reserves^35^. Maize embryos dry weight consists of 50% lipid reserves^36^, however MDA measurements did not reveal that germination at 10 °C induced increased lipid peroxidation nor that the amount of this compound differed between cold tolerant and sensitive hybrids at low temperature (Fig. 2a). Due to the very low relative amount of phospholipids compared to the one of triacylglycerols in maize embryos, MDA measurements cannot reveal any damage at this level, although cold stress has often been mentioned to target directly membrane properties, as reviewed by Yadav^37^. Therefore negative effects of cold imbibition on membranes were assessed by comparing electrolyte leakage in hybrids A and B (Fig. 2b). After imbibition at a non-penalizing temperature (18 °C), leakage in hybrid B was higher than in hybrid A which could result from hybrid-specific membrane properties. However at 10 °C leakage significantly increased in seeds of hybrid B, but not in the ones of hybrid A. Seed imbibition is a particularly cold-sensitive step of the germination process. During germination membranes reorganize from a glassy and static state to a gel and fluid structure^21^. Imbibition is associated with membrane repair that leads to a regulated and moderated leakage of electrolytes^38^. This process allows the recovery of membrane functionalities. Some authors highlighted that environmental stress, and particularly low temperature, led to dramatic and sudden electrolytes leakage during imbibition^39^. This rapid water uptake can cause imbibitional injuries resulting from a disruption of membrane remodeling^40, 41^. Results presented in Fig. 2b confirmed the trend of hybrid B to be more sensitive to cold because of a lack of membrane stability during imbibition at 10 °C.

With regards to the effect of chilling on electrolyte leakage, we thus compared phospholipidome remodeling in seeds of cold tolerant and sensitive hybrids during their imbibition at 10 °C. The very powerful method used in this study (Mass Spectrometry using the Multiple Reaction Monitoring) provided a high resolution of membrane remodeling. Indeed, quantification of species from each phospholipid class allowed to precisely address changes in membrane composition in response to cold stress and to demonstrate that phospholipid profiles of the two hybrids differed both in dry embryos and after imbibition at 10 °C and 18 °C (Fig. 3). Distribution of phospholipid classes including PE, PC, PI and PG, is very similar to the one shown by Harrabi *et al*. 42 who provided the phospholipid composition in different corn seed tissues. The PCA shown on Fig. 4 demonstrated that differences in phospholipid composition between hybrids were rather associated with an effect of cold imbibition than related to a genetic factor. Imbibition at 18 °C did not fully discriminate the lipid species. In addition, the loading of the principal component 1 (Supplemental Table S1) also indicated that the discrimination of the two hybrids after cold imbibition relied more on fatty acids composition *per se* than on the phospholipid class. Additional information was provided by K-means clustering analysis shown on Fig. 5 which revealed specific profiles of lipids for seeds of sensitive and tolerant hybrids during cold imbibition. We demonstrated that linoleic acid is the major phospholipid fatty acid which abundance decreased during cold-imbibition in the sensitive hybrid (cluster 1, Fig. 5). This suggests that the amount of C18:2 can be related to cold-tolerance in maize seeds. Linoleic acid accumulation has already been shown to play a role in cold acclimation in potato^43^ or rice^44^. In contrast cold imbibition was associated with an increase in saturation of membrane lipids in the sensitive hybrid (cluster 2, Fig. 5), as previously demonstrated in rice, for example (Cruz *et al*.^45^). The statistical trends were confirmed by analysis and quantification of individual fatty acids (Fig. 6). Whatever the membrane lipid class, cold sensitivity was associated with a decrease in unsaturation grade through an accumulation of stearic (C18:0) and oleic (C18:1) acids at the expense of linoleic acid (C18:2) whereas the amounts of palmitic acid (C16:0), palmitoleic acid (C16:1) and hexadecatrienoic acid (C16:3) were not modified by cold treatment (Fig. 6 and Supplemental Figures S1, S2 and S3). In consequence, embryos of the tolerant hybrids were characterized by a high DBI for all phospholipid classes. Zheng *et al*.^46^ recently highlighted the response of a cold exposure on DBI and ACL (Acyl Carbon Length) of glycerophospholipids in *Arabidopsis thaliana* and rice and the consequences of these changes on the membrane structure and properties.

Membrane unsaturation is thought to have a major effect on membrane permeability properties, especially during the critical step of seed imbibition when phospholipids remodeling occurs. Indeed, unsaturation increases membrane fluidity which is needed for its function of signaling surface^47, 48^. Cold can interact with this reorganization and thus impact membrane properties, as it is shown here with electrolyte leakage measurements (Fig. 2). Our results suggest that cold induced a membrane disorganization in seeds of the sensitive hybrid, which failed to increase phospholipids acyl chain unsaturation when compared to seeds of the tolerant hybrid, thus leading to an increased electrolyte leakage. This caused a delay in germination whereas seeds of the cold-tolerant hybrid used cellular mechanisms to adapt to this environmental stress. Tolerant hybrids can increase their phospholipid unsaturation level to enhance the membrane fluidity in response to cold^46^.

Since FA unsaturation changed in response to cold imbibition, one could expect significant changes in expression of genes coding for desaturases which introduce a second double bond into C18:1 fatty acids, *i.e*. *fad2* and *fad6*. Analysis of gene expression revealed that cold imbibition induced a transient accumulation of *fad2* transcripts in both embryos of tolerant and sensitive hybrids but that 24 h imbibition at 18 °C also led to an over-expression of *fad2*. This is not supporting a major role of this ER desaturase in the observed changes in FA pattern or this suggests that cold does not regulate transcription but activity of this enzyme (Fig. 7). Indeed although fatty acid unsaturation is known to be related to activities of fatty acid desaturases this is not necessarily related to a transcriptional regulation^49^. Similar results have been observed in *Arabidopsis thaliana* shoots^50^ and in soybean seeds^51^ where *fad2* genes were not activated by low temperature and Matteucci *et al*.^52^ demonstrated that cold acclimation of olive drupes relied on FAD post-transcriptional regulation. Conversely other studies demonstrated that transcription of ω-6 desaturase was up-regulated by cold in cotton^53^, citrus^54^ and cucumber^55^. In contrast, cold induced a rapid and transient expression of the plastidial desaturase *fad6* in embryos of the tolerant hybrid only (Fig. 7c). This is suggesting that the accumulation of C18:2 observed in PG of seeds of hybrid A in response to cold (Supplemental Figure S3) would result from the over-expression of *fad6* in the early steps of seed imbibition. At last expression of *ssi2* was higher during cold imbibition than in dry embryos whereas it did not change significantly in the sensitive hybrid (Fig. 7a). However this effect may have a genetic component since in non-stressful conditions, *i.e*. after 24 h at 18 °C, the expression of *ssi2* was also stimulated in seeds of the tolerant hybrid. Ding *et al*.^56^ demonstrated that drought stress, ABA and low temperature altered the expression of *sad*, an analogue of *ssi2* in *Camellia sinensis* leaf. Cold has also been shown to up-regulate *ssi2* in potato^43^, in *Brassica napus* hypocotyl^57^ and in shoots of cassava^58^. De Palma *et al*.^59^ also showed that overexpression of *ssi2* in transformed potato enhanced the cold tolerance by decreasing the ratio of saturated to unsaturated fatty acids in phospholipids which was related to the prevention of membrane damages. Altogether the gene expression data presented here highlighted a complex mechanism of regulation of FA desaturation in response to cold, associating transcriptional and most likely post-transcriptional issues in both plastid and endoplasmic reticulum.

The present work brings a comprehensive model of the cellular basis of response to cold stress at the germination stage in maize seeds taking advantage of the lipidomic approach. We clearly demonstrate that the ability of maize seeds to germinate rapidly at low temperature requires phospholipid remodeling in order to prevent loss of membrane properties. Besides the fundamental knowledge provided here, our findings should help to design future breeding programs in order to create novel hybrids whose seeds will be able to cope with low temperature regime at the time of the sowing period.

## Methods

### Plant material

Seeds of hybrids A and B of *Zea mays* were provided by Limagrain Europe. They have been harvested in 2012 in South of France and stored at 20 °C and 50% relative humidity until experiments.

### Germination assays

Germination assays were carried out using whole grains at 5, 10, 15 or 18 °C in darkness, in 3 replicates of 25 grains. Seeds were placed in Petri dishes (9 cm diameter) on a layer of cotton wool imbibed with deionized water. Grains were considered as germinated when the radicle elongated by approximately 3 mm. Results presented correspond to the means of the germination percentages obtained with 3 replicates ± SD. Germination indicators have been calculated according to Ni & Bradford^60^. T50 is the time (in h) needed to obtain 50% of the final germination. Thermal time is the cumulative degrees-hours (°C.h) required to reach 50% of the germination at suboptimal temperatures.

### Malondialdehyde measurements

Lipid peroxidation was evaluated by measuring malondialdehyde (MDA) content of 5 embryos of dry or imbibed seeds, according to the method of Heath & Packer^61^ with slight modifications. Embryos excised from seeds were ground in 5 mL distilled water and homogenized with an equal volume of 0.5% (w/v) 2-thiobarbituric acid and 20% (w/v) trichloroacetic acid. The homogenate was incubated at 95 °C for 30 min and then centrifuged at 16 000 *g* for 30 min. The supernatant was used for MDA determination. Results are expressed as nmol.g^−1^ of dry weight and correspond to the means of measurements carried out with 3 extracts ± SD.

### Electrolyte leakage measurements

Membrane permeability has been studied on whole grain (embryo + endosperm + pericarp), with 5 grains per triplicate for each hybrid after 24 h imbibition at 10 °C or 18 °C. Conductivity was measured after soaking the seeds for 1 h in 10 mL of distilled water at 10 or 18 °C with a conductivity meter (Consort, K220). Total conductivity was estimated after boiling seeds at 100 °C during 10 min and results are expressed as % of the measured leakage to the total leakage.

### Total lipid extraction

Embryos (5 per replicate) were excised from seeds imbibed at 10 °C or 18 °C for 24 h in the dark and ground to a fine powder in liquid nitrogen with pre-chilled mortar and pestle. The powder was added to 3 mL of hot (70 °C) isopropanol. After 5 min of mild agitation, 3 mL of chloroform was added. Then 3 mL of 0.9% (w/v) NaCl was added to initiate phase separation. After a night at 4 °C, lipid phase was taken and evaporated under nitrogen stream. Lipids were re-suspended in 400 µL chloroform.

### Phospholipid analysis

Lipids were analysed by mass spectrometry in the multiple reaction monitoring (MRM) mode in which tandem mass spectrometry is implemented with collision-induced dissociation (CID). The method was similar to the one described by Rainteau *et al*.^24^ which has been developed for analysing plant lipids. The distinct glycerophospholipid classes were eluted successively as a function of the polar head group by an Agilent 1100 HPLC system equipped with a 250 mm × 4 mm (length × internal diameter) 5 µm Lichrospher silica column at 65 °C. Two types of separation were performed. For the analysis of PC, PI, and galactolipids the mobile phase consisted of A: hexane/isopropanol/water (628:348:24, v/v) supplemented with 10 mg/L ammonium formate and B: isopropanol/water (850:146, v/v) supplemented with 10 mg/L ammonium formate. The percentage of B increased linearly from 0% to 40% in 45 min and then to 100% in 3 min. The flow rate of the mobile phase was 300 µL/min^24, 25^. For the analysis of PG and PE, the mobile phase consisted of A: ammonium acetate 10 mM pH 5.3/acetonitrile (5:95, v/v) and B: ammonium acetate 10 mM pH 5.3/acetonitrile (50:50, v/v). The percentage of B increased linearly from 0% to 20% in 45 min and then to 95% in 1 min. The percentage in B was maintained at 95% for 5 min and then decreased to 0% in 1 min. The flow rate of the mobile phase was 400 µL/min. Eluted lipids were continuously injected to tandem mass spectrometer (QTrap2000, ABSciex) and the MRM analysis was performed using the same parameters as the ones described in Rainteau *et al*.^24^ and acquired during the entire HPLC run.

### RNA extraction and qRT-PCR

Ten embryos were ground in liquid nitrogen and total RNA was extracted from 100 mg of the resulting powder by a hot phenol procedure according to Verwoerd *et al*.^62^. DNAse treatment and reverse transcription were performed with 2 µg total RNA as described by Leymarie *et al*.^63^. Q RT-PCR was performed in a 15 µL total volume of Maxima SYBR Green/ROX qPCR Master Mix (Thermo Scientific, US) following addition of the cDNA and the gene-specific primers (Supplemental Table S2). Relative expressions were calculated according to Hellemans *et al*.^64^ with 3 reference genes: a cyclin-dependant kinase (*cdk*, ID: GRMZM2G149286), a hypothetical protein (*unknown*, ID: GRMZM2G047204) and a 2OG-Fe oxygenase family protein (*2og-fe*, ID: GRMZM2G114098) described as housekeeping genes in maize by Lin *et al*.^65^. *fad2* and *fad6* primers were designed from cDNAs of genes expressed in embryos provided by Mikkilineni & Rocheford^66^ and *ssi2* primers from cDNA isolated by Soderlund *et al*.^67^. Gene expression values are provided in arbitrary units with the value of 100 corresponding to the dry seeds, which was used as control sample for normalization^68^.

### Statistical analysis

Software R version 3.1.2 was used for PCA and Tukey tests. MultiExperiment Viewer version 4.9.0 was used for the K-means clustering.

## Electronic supplementary material

Supplementary Information
