# Supplementary material for: Chilling temperature remodels phospholipidome of Zea mays seeds during imbibition
Source: Sci Rep. 2017 Aug 21;7:8886. doi: 10.1038/s41598-017-08904-z (PMC5566375; doi:10.1038/s41598-017-08904-z)
Supplement: Supplementary file 1 — Supplementary Information [file 41598_2017_8904_MOESM1_ESM.pdf]

Chilling temperature remodels phospholipidome of *Zea mays* seeds during imbibition

Agathe Noblet, Juliette Leymarie & Christophe Bailly

Supplementary Information

Supplemental Table S1: Loadings of PCA of membrane compositions.

SPECIES	PC1	PC2
**PG.16.0.18.2**	0,17529876	0,02904078
**PE.18.2.18.2**	0,17409219	-0,00625549
**PI.18.2.18.2**	0,17006974	-0,01376637
**PC.18.2.18.2**	0,16156008	0,05729148
**PI.16.0.18.2**	0,15342682	0,10552665
**PC.16.0.18.2**	0,15203174	-0,02593991
**PG.18.2.18.2**	0,14847535	-0,13018102
**PE.16.0.18.2**	0,14116284	0,12819226
**PI.18.3.18.2**	0,12049219	0,12148553
**PI.18.2.18.1**	0,10159119	-0,19480106
**PG.16.1.18.2**	0,10034802	-0,15845692
**PC.18.3.18.2**	0,06439632	0,08304756
**PI.18.3.18.3**	0,05835011	0,08506985
**PI.16.3.18.2**	0,05824309	0,11823693
**PI.16.1.18.3**	0,05682067	0,02840061
**PG.16.3.18.2**	0,05672016	-0,12001968
**PE.16.3.18.1**	0,05345882	0,08651567
**PI.16.1.18.2**	0,03409181	0,08448335
**PG.18.3.18.2**	0,03374481	0,19831261
**PG.18.0.18.2**	0,03339112	0,14315211
**PE.16.1.18.3**	0,03295768	0,13412994
**PC.16.1.18.3**	0,02660858	0,08301636
**PC.16.3.18.2**	0,02396509	-0,21178764
**PE.18.3.18.3**	0,02016405	-0,22544126
**PE.16.3.18.0**	0,01970213	0,12546044
**PC.18.2.18.1**	0,01915402	-0,2190797
**PE.18.3.18.2**	0,0150789	-0,06977544
**PG.18.2.18.1**	0,01064454	-0,23820162
**PE.16.3.18.2**	0,00339087	-0,04712839
**PI.16.3.18.0**	0,00311872	-0,16676066
**PG.16.3.18.3**	-0,00150129	-0,14392364
**PI.16.3.18.1**	-0,00739175	-0,04877989
**PC.18.0.18.2**	-0,00819855	-0,07564785
**PG.18.3.18.3**	-0,01128453	0,21901518
**PG.16.3.18.1**	-0,01836378	-0,14899014
**PC.16.1.18.2**	-0,01960234	0,18908085
**PC.16.3.18.3**	-0,0207869	0,06790667
**PC.18.0.18.3**	-0,02445039	-0,04591027
**PG.16.3.18.0**	-0,02778667	-0,05978473
**PE.16.1.18.2**	-0,036323	0,19042596
**PC.16.3.18.1**	-0,03831318	0,14296623
**PG.16.0.18.3**	-0,04715527	0,14275853
**PI.16.3.18.3**	-0,04979294	-0,00454658
**PE.18.0.18.2**	-0,05260517	0,19223277
**PE.16.3.18.3**	-0,05810517	-0,15720742
**PC.18.3.18.3**	-0,06196666	-0,04522984
**PG.16.1.18.3**	-0,07546762	-0,06316509
**PC.16.3.18.0**	-0,0807742	0,06722759
**PI.18.0.18.3**	-0,0809209	-0,00609631
**PE.18.0.18.3**	-0,0862073	-0,07645999
**PE.16.0.16.0**	-0,10455234	-0,15830078
**PG.16.1.18.1**	-0,10849776	-0,043949
**PC.16.0.16.0**	-0,11191241	0,12399185
**PI.16.1.18.1**	-0,11861329	0,06099325
**PI.18.1.18.3**	-0,12008564	-0,01068549
**PI.18.0.18.2**	-0,12063994	0,08550755
**PE.18.2.18.1**	-0,1228895	-0,16672921
**PI.16.0.18.3**	-0,1242259	0,12852904
**PE.16.1.18.1**	-0,12776439	-0,00822221
**PC.16.0.18.3**	-0,13550462	0,07585134
**PG.18.0.18.3**	-0,13834358	0,0677913
**PC.18.0.18.1**	-0,14130074	-0,12110763
**PI.16.0.16.0**	-0,14616528	-0,05094051
**PI.18.0.18.1**	-0,14825037	-0,13686731
**PG.16.0.16.0**	-0,14941512	0,12728107
**PE.16.0.18.3**	-0,15261454	0,03921423
**PC.16.0.18.1**	-0,16469022	0,06566155
**PE.18.1.18.3**	-0,16511363	-0,0544864
**PC.18.1.18.3**	-0,16574035	0,04687218
**PG.18.1.18.3**	-0,16747053	0,00997433
**PC.16.1.18.1**	-0,16804182	-0,00125752
**PI.18.1.18.1**	-0,17235626	-0,01125375
**PE.18.0.18.1**	-0,17323712	-0,04038533
**PG.18.1.18.1**	-0,17345996	-0,011749
**PG.16.0.18.1**	-0,17353249	0,04496531
**PG.18.0.18.1**	-0,17431842	-0,02866872
**PE.16.0.18.1**	-0,1758361	0,04091707
**PI.16.0.18.1**	-0,17773599	-0,01393308
**PC.18.1.18.1**	-0,17840719	-0,01439938
**PE.18.1.18.1**	-0,17905749	0,01821976

Supplemental Table S2: Genes and primer sequences used for qPCR analysis. *fad2*, *fad6* and *ssi2* are target genes. *unknown, cdk* and *2og-fe* are housekeeping genes.

Putative gene	accession	Forward/ Reverse	Primer sequence
fad2	DQ496227	Forward	ATGGTGCCCTACTTCTCGTG
		Reverse	GCCGACCGGGTTGTTGTA
fad6	100101547	Forward	GTATTGTCGGCACAGGTGGA
		Reverse	ACGCTACGTGCTTTCCTACT
Ssi2	100192652	Forward	GAAACCCATTGCGACTGCTT
		Reverse	GAGACGGCACAAGGCAAAAC
Unknown	GRMZM2G047204	Forward	GCGTGCTCTTTCGTCAGATGTG
		Reverse	CCTACTGTTGGCTGGAGACTGG
cdk	GRMZM2G149286	Forward	CCGTCATCGCCTCACGAAGAG
		Reverse	AGAGCCTGCCTTACGGAATTGG
2og-fe	GRMZM2G114098	Forward	CGTCCTGTCTCCGCTTCAAGTG
		Reverse	GCCACTGCCTGCTATTGTCCAC

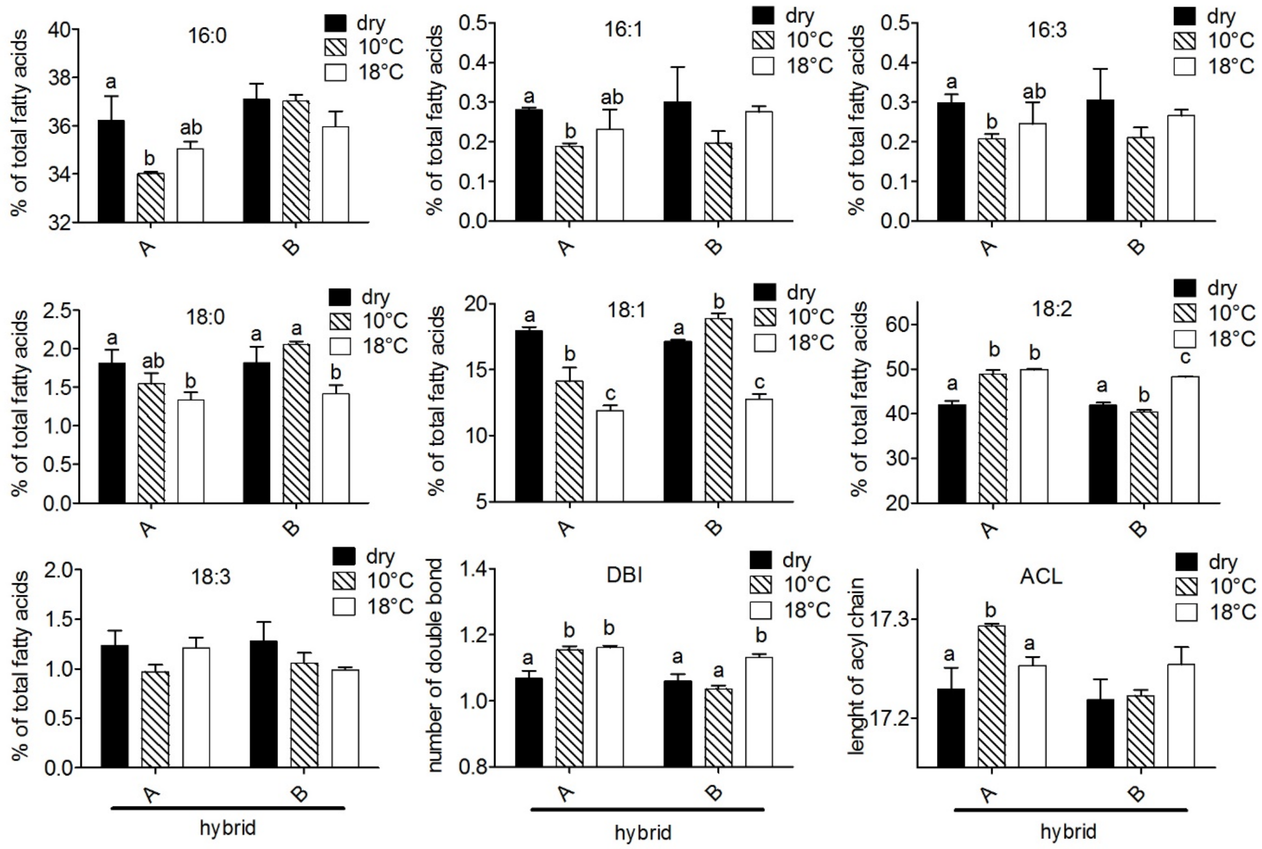

Figure S1. Fatty acid composition of phosphatidylinositol (PI). Proportion (in %) of fatty acid chains in dry and imbibed embryos of hybrids A and B. DBI: double bond index, ACL: acyl carbon length. Different letters denote significatively different mean values at $P < 0.05$ according to Tukey's multiple range test. Absence of letters indicates that means are not significantly different.

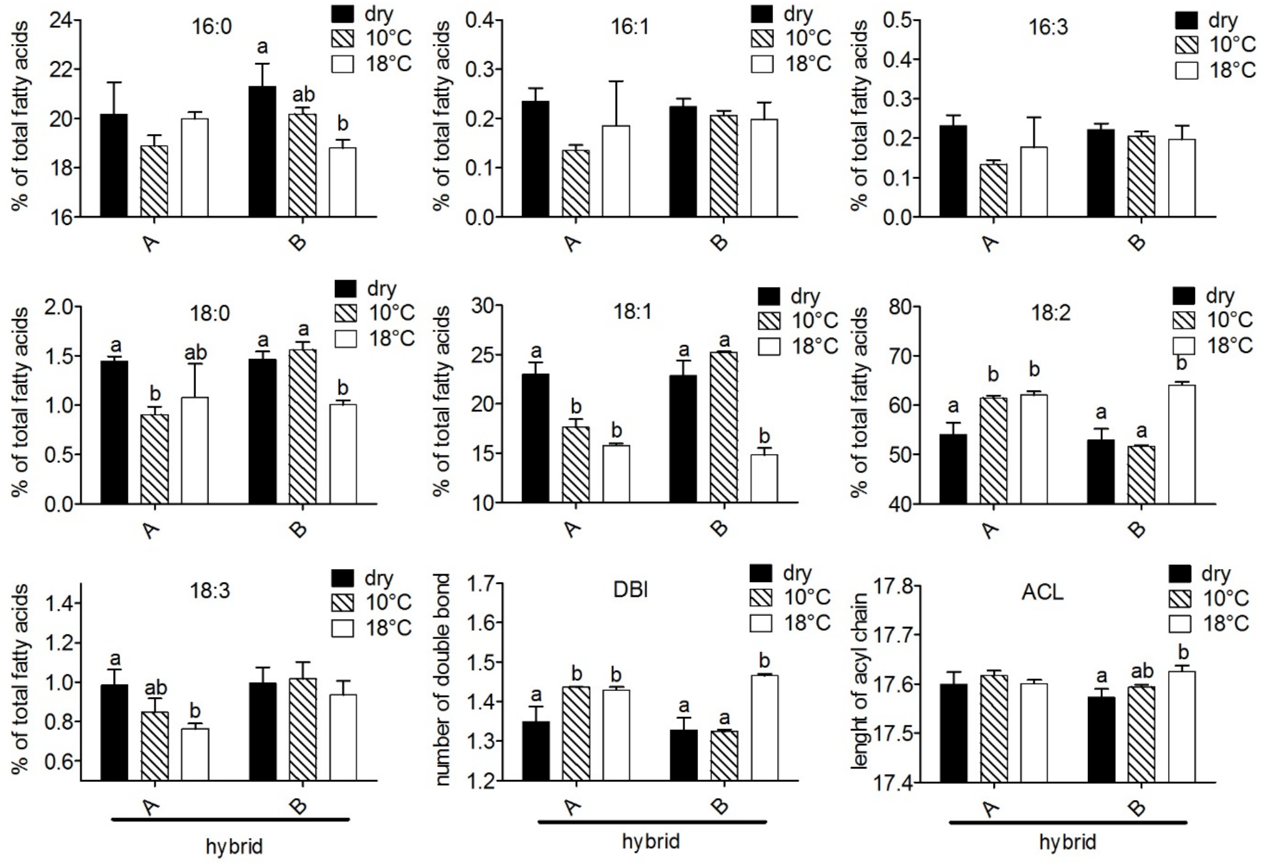

Figure S2. Fatty acid composition of phosphatidylethanolamine (PE). Proportion (in %) of fatty acid chains in dry and imbibed embryos of hybrids A and B. DBI: double bond index, ACL: acyl carbon length. Different letters denote significatively different mean values at $P < 0.05$ according to Tukey's multiple range test. Absence of letters indicates that means are not significantly different.

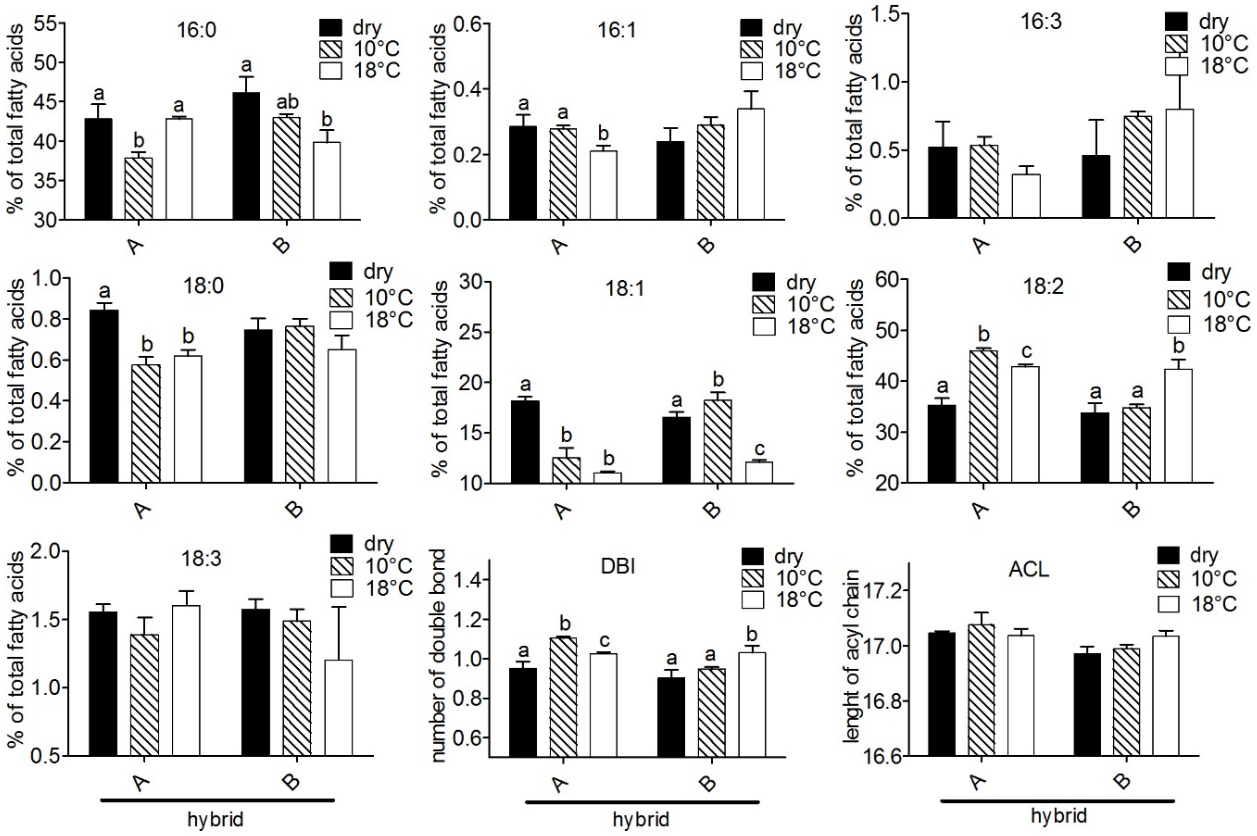

Figure S3. Fatty acid composition of phosphatidylglycerol (PG). Proportion (in %) of fatty acid chains in dry and imbibed embryos of hybrids A and B. DBI: double bond index, ACL: acyl carbon length. Different letters denote significatively different mean values at $P < 0.05$ according to Tukey's multiple range test. Absence of letters indicates that means are not significantly different.
